# ﻿Revision of the genus *Charitoprepes* Warren (Lepidoptera, Crambidae), with the description of a new species from China

**DOI:** 10.3897/zookeys.1149.98065

**Published:** 2023-02-23

**Authors:** Shi-Qi Huang, Xi-Cui Du

**Affiliations:** 1 College of Plant Protection, Southwest University, Chongqing, China Southwest University Chongqing China

**Keywords:** *Charitoprepesaciculata* sp. nov., genitalia, Pyraloidea, Spilomelinae

## Abstract

The genus *Charitoprepes* is revised based on morphological characteristics, and *Charitoprepesaciculata***sp. nov.** is described as new from China. Additionally, the female genitalia of *C.lubricosa* are described for the first time based on new material. The differences among species of this genus are diagnosed, along with images of adults and their genitalia.

## ﻿Introduction

The genus *Charitoprepes* was erected by Warren (1896) with *Charitoprepeslubricosa* Warren, 1896 from India as the type species. Hampson (1896) synonymized *Charitoprepes* with *Heterocnephes* Lederer, 1863, which was followed by other authors for more than 100 years. *Charitoprepes* was treated as valid genus by Kim et al. (2014), with *C.lubricosa* as the only species. Mally et al. (2019) transferred *Heterocnephesapicipicta* to *Charitoprepes* based on morphological characteristics and, until now, this genus only contained two known species (Nuss et al. 2023). The genus is distributed in China, India, Japan, and South Korea (Inoue 1963; Wang et al. 2003; Choi 2010; Kim et al. 2014).

*Charitoprepes* species are easily distinguished from those of other genera in having an elongated elliptical black patch at the apex of the greyish brown forewings. Species in this genus are externally very similar, but they can be distinguished by their genitalia. In this study, the morphological characteristics of this genus are revised, and one new species is described from China.

## ﻿Materials and methods

The specimens were collected using a light trap and killed with ethyl acetate or ammonium hydroxide. The specimens, including the type material of the new species, are deposited in the College of Plant Protection, Southwest University, Chongqing, China (**SWU**). The corresponding author examined the type specimen of *Charitoprepeslubricosa* deposited in Natural History Museum, London, United Kingdom (**NHMUK**). Genitalia preparation mainly follows Li and Zheng (1996). Images of the adults were photographed using a digital camera (Nikon P7700), and images of the genitalia were captured with a digital camera (Leica DFC 450) attached to a digital microscope (Leica M205 A). The terminology mainly follows Maes (1995) and Mally et al. (2019).

## ﻿Taxonomy

### ﻿ Charitoprepes

Taxon classificationAnimaliaLepidopteraCrambidae

Warren, 1896

94419954-E4FD-5889-8CE4-4C82EBD18DEA

Charitoprepes Warren, 1896: 136. Type species: Charitoprepeslubricosa Warren, 1896, by original designation.

#### Diagnosis.

This genus is distinguished by the greyish-brown body and wings; the forewing with an elongated, elliptical, black patch at the apex. This genus can be distinguished from *Heterocnephes* by its labial palpi bent and upturned normally, the corpus bursae with two thin, band-like signa present or absent. In *Heterocnephes*, however, the second segment of labial palpi is inflated and nearly oblong, along with its third segment protruded forward (Wang 1980), the corpus bursae has two round signa, and the valva is broader than that of *Charitoprepes*.

#### Generic characteristics.

**Figure 1.**
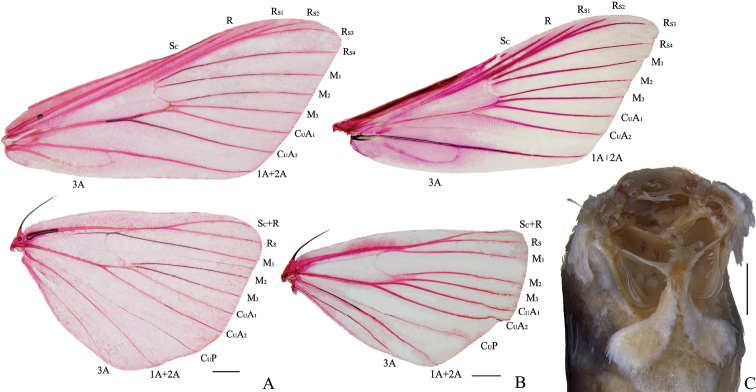
Wing venation and tympanal organs of *Charitoprepes***A, C***C.lubricosa***B***C.aciculata* sp. nov. Upper is forewing, lower is hindwing. Scale bars: 1.0 mm (**A, B**); 0.5 mm (**C**).

***Adult*.** Body and wings greyish brown. Frons rounded. Antenna filiform, with sparse cilia ventrally. Labial palpi bent and upturned. Maxillary palpi filiform. Forewing with orbicular and discoidal stigma present, an elongated, elliptical, black patch along costa at apex; length of cell approximately half of wing; discocellulars arcuately incurved; R_S1_ very close to R_S2+S3_; R_S2_ anastomosed with R_S3_ approximately three-fifths beyond cell; M_2_, M_3_ and CuA_1_ originating from posterior angle of the cell and uniformly spaced at base. Hindwing with length of cell half of wing; discocellulars strongly, arcuately incurved; Rs anastomosed with Sc+R at long distance; M_1_ and Rs shortly stalked at base beyond cell; M_2_, M_3_ and CuA_1_ originating from posterior angle of cell (Fig. 1A, B). Legs shiny white. Middle tibia with distal inner spur approximately twice length of outer spur; hind tibia with inner proximal spur approximately triple length of outer proximal spur, and inner distal spur approximately twice length of outer distal spur. Tympanal organs with fornix tympani surface projecting from the tympanic frame. Praecinctorium strongly bifid (Fig. 1C).

***Male genitalia*.** Uncus long and thin, with the distal part swollen and covered with minute setae. Valva broad. Fibula spine-like and downcurved. Sacculus sclerotized, with an apical triangular process overlapping with the fibula. Saccus broad and rounded, tapered terminally. Cornutus present and diverse.

***Female genitalia*.** Apophyses anteriores as long as apophyses posteriores, or longer. Ductus bursae varies from short and broad to long and thin. Corpus bursae elliptical or oval. A pair of thin, band-like signa present or absent.

#### Distribution.

**Figure 2.**
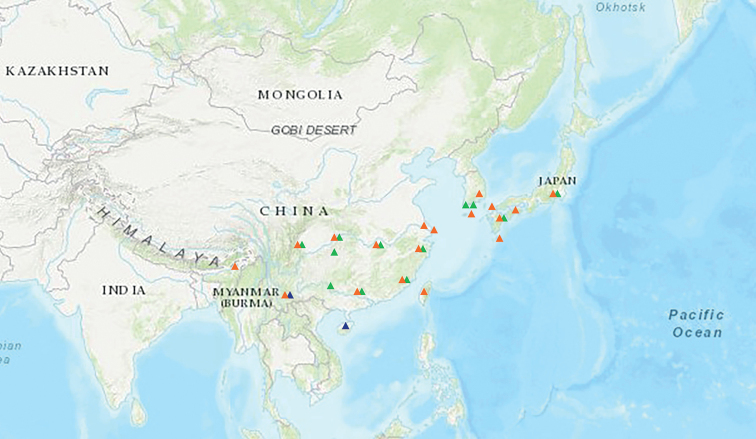
Distribution of *Charitoprepes* spp. (*C.lubricosa*: orange triangles; *C.apicipicta*: green triangles; *C.aciculata* sp. nov.: blue triangles).

China, India, Japan, South Korea (Fig. 2).

#### Remarks.

According to Kim et al. (2014), this genus can be identified by an elongated, elliptical, black patch at apex of forewing and dark brown, discoidal stigma on the hindwing. In *C.aciculata* sp. nov., however, the discoidal stigma on the hindwing is absent. Therefore, the morphological characteristics of *Charitoprepes* have been revised in this study, with supplementary wing venation and genitalia characteristics.

### ﻿Key to species of *Charitoprepes* based on morphology and genitalia

**Table:** 

1	Discoidal stigma absent on hindwing; phallus slender and extremely elongated, with an elongated, needle-like cornutus	***C.aciculata* sp. nov.**
–	Discoidal stigma present on hindwing; phallus short and stout	**2**
2	Phallus with a spicate cornutus	***C.lubricosa***
–	Phallus with two fusiform cornuti	***C.apicipicta***

### ﻿ Charitoprepes lubricosa

Taxon classificationAnimaliaLepidopteraCrambidae

Warren, 1896

52454305-8A8F-5EAC-9ECE-2E9CB7A6B668

**Figure 3.**
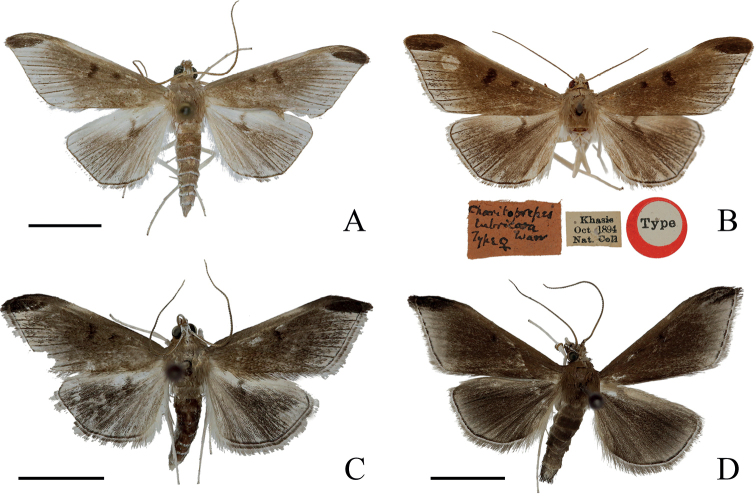
Adults of *Charitoprepes* spp. **A***C.lubricosa*, male **B***C.lubricosa*, female, type (NHMUK) **C***C.apicipicta*, male **D***C.aciculata* sp. nov., male, holotype. Scale bars: 0.5 cm.

**Figure 4.**
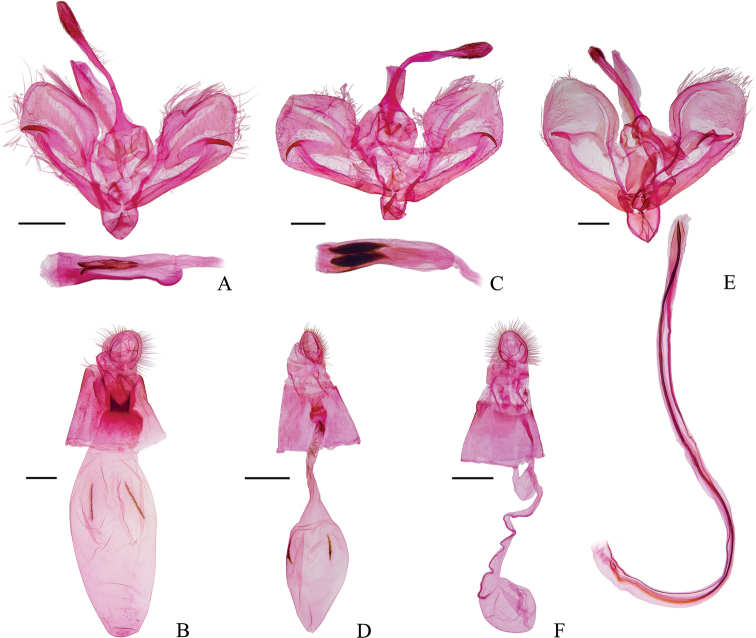
Genitalia of *Charitoprepes* spp. **A, B***C.lubricosa***C, D***C.apicipicta***E, F***C.aciculata* sp. nov. **A** male, genitalia slide no. HSQ22166 **B** female, genitalia slide no. HSQ22163 **C** male, genitalia slide no. HSQ22161 **D** female, genitalia slide no. HSQ22167 **E** male, paratype, genitalia slide no. HSQ22164 **F** female, paratype, genitalia slide no. HSQ22168. Scale bars: 0.5 mm (**A, C, E**); 1.0 mm (**B, D, F**).

Figs 1A , 3A, B , 4A, B

Charitoprepes lubricosa Warren, 1896: 136. Type locality: India (Meghalaya). Type depository: NHMUK.
Heterocnephes lubricosa : Hampson, 1896: 265.

#### Material examined.

***Holotype***, ♀ India, Khasis, X. 1894, Nat. Coll. (NHMUK). **Additional material.** China, **Chongqing Municipality**, 4 ♂♂, 2 ♀♀, Chengkou County, Dongan Town, Xingtian Village, 1300 m elev., 30 June 2013, Gui-Qing He & Li-Jun Xu leg., Genitalia slide no. HSQ22163 ♀; 1 ♂, Jinfo Mountain, 696 m elev., 18 May 2017, Ji-Ping Wan & Qiu-Long Yang leg., Genitalia slide no. HSQ22166 ♂; 2 ♂♂, Simian Mountain, 900 m elev., 18 July 2012, Gui-Qing He leg., wing slide no. HSQ22003; **Guangdong Prov.**, 1 ♂, Nanling Nature Reserve, Babao Reserve Station, 1070 m elev., 23 August 2010, Xi-Cui Du leg.; **Sichuan Prov.**, 1 ♀, Xuyong County, Guandou Town, 501 m elev., 29 August 2013, Li-Jun Xu leg.; **Yunnan Prov.**, 1 ♂, Xishuangbanna Dai Autonomous Prefecture, Yaoqu Town, 780 m elev., 26 May 2015, Man-Fei Tao leg., Genitalia slide no. HSQ22160 ♂; **Zhejiang Prov.**, 2 ♂♂, 1 ♀, Tianmu Mountain, 400 m elev., 26 July 2011, Xi-Cui Du leg.

#### Description.

**Adult** (Fig. 3A, B). Body and wings pale greyish brown. Forewing length 10.0–14.0 mm, wingspan 21.0–29.0 mm. Frons pale greyish brown, white laterally; vertex white. Antenna brown, scape white ventrally. Labial palpi with first segment white, second and third segments brown. Maxillary palpi white, brown near apex. Patagium, tegula and pale greyish brown. Fore and hind wings with terminal area pearly grey. Forewing greyish brown along veins, orbicular stigma and discoidal stigma conspicuous and dark brown; middle third of costa pearly grey. Hindwing with dark brown, discoidal stigma. Veins towards margin finely dark on fore and hind wings. Cilia brown, with a white basal line. Legs shiny white, epiphysis orange-yellow. Abdomen pale greyish brown, pale grey ventrally.

***Male genitalia*** (Fig. 4A). Valva square, with sparse setae, narrowed at base. Fibula well developed. Saccus broad. Phallus stout, with a spicate cornutus.

***Female genitalia*** (Fig. 4B). Apophyses anteriores slightly longer than apophyses posteriores. Antrum sclerotized, developed. Ductus bursae short, about half as wide as corpus bursae. Corpus bursae large, elongate elliptical, with a pair of narrow, longitudinal, band-like signa.

#### Distribution.

China (Chongqing, Fujian, Guangdong, Hubei, Jiangsu, Shanghai, Sichuan, Taiwan, Yunnan, Zhejiang), India, Japan, South Korea.

#### Remarks.

The female genitalia of this species are described for the first time.

### ﻿ Charitoprepes apicipicta

Taxon classificationAnimaliaLepidopteraCrambidae

(Inoue, 1963)

E53F7E31-323F-5FB4-9EEF-F0C9ACBC6ED3

Figs 3C , 4C, D

Heterocnephes apicipicta Inoue, 1963: 109. Type locality: Japan (Honshu).
Charitoprepes apicipicta : Mally et al. 2019: 141.

#### Material examined.

China, **Chongqing Municipality**, 2 ♂♂, 2 ♀♀, Wushan County, Dangyang Town, Wulipo Nature Reserve, 396 m elev., 19 April 2021, Hong Zhao & Jin-Hang Han leg., Genitalia slide no. HSQ22165 ♂; 4 ♂♂, Wuxi County, Yintiaoling Nature Reserve, Hongqihegou, 1118 m elev., 21 June 2022, Ci Tang & Xin-Lei Xue leg.; 1 ♂, Simian Mountain, 1280 m elev., 11 August 2011, Gui-Qing He & Li-Fang Song leg., Genitalia slide no. HSQ22149 ♂; **Guangdong Prov.**, 1 ♂, 1 ♀, Nanling Nature Reserve, Babao Reserve Station, 1070 m elev., 23 August 2010, Xi-Cui Du leg.; **Guangxi Zhuang Autonomous Region**, 1 ♂, 2 ♀♀, Nonggang Nature Reserve, 188 m elev., 25 July 2011, Gui-Qing He leg.; 2 ♂♂, Nonggang Nature Reserve, 170 m elev., 21 August 2020, Lin-Lin Yang leg., Genitalia slide no. HSQ22161 ♂; 1 ♀, Nonggang Nature Reserve, 170 m elev., 21 August 2020, Lin-Lin Yang leg., Genitalia slide no. HSQ22167 ♀; **Guizhou Prov.**, 3 ♂♂, Kuankuoshui Nature Reserve, 800 m elev., 11 August 2010, Xi-Cui Du leg.; **Zhejiang Prov.**, 6 ♂♂, 3 ♀♀, Jiulong Mountain, 6 August 2011, Xiao-Bing Fu leg.; 1 ♂, 3 ♀♀, Tianmu Mountain, 400 m elev., 2 August 2011, Xi-Cui Du & Xiao-Bing Fu leg.

#### Diagnosis.

This species is very similar to *C.lubricosa* in appearance, but its greyish brown wings and body are darker than those of *C.lubricosa*. It also can be distinguished by the stout phallus, which has two fusiform cornuti decorated with numerous minute spines, and the ductus bursae, which is elongated and far more slender than that of *C.lubricosa*.

#### Description.

***Male genitalia*** (Fig. 4C). Valva square, with sparse setae. Saccus broad. Phallus stout, two fusiform cornuti decorated with numerous minute spines (Inoue 1963).

***Female genitalia*** (Fig. 4D). Apophyses anteriores as long as apophyses posteriores. Antrum sclerotized. Ductus bursae long, membranous. Corpus bursae elliptical, taper distally, with a pair of narrow, longitudinal, band-like signa.

#### Distribution.

China (Chongqing, Fujian, Guangdong, Guangxi, Guizhou, Hubei, Sichuan, Zhejiang), Japan, South Korea.

#### Remarks.

There are occasionally some spines scattered in the ductus bursae of female specimens, which suggests that the cornuti in the male genitalia are deciduous (Fig. 4D).

### ﻿ Charitoprepes aciculata sp. nov.

Taxon classificationAnimaliaLepidopteraCrambidae

BC7BFA7E-C75B-5159-97FD-4FC158326B25

https://zoobank.org/F62E09A4-F279-468A-A51C-F8ACA9149117

Figs 1B , 3D , 4E, F

#### Type material.

***Holotype*.** ♂, pinned, with genitalia on a separate slide, China, **Hainan Prov.**, Wuzhi Mountain, 18°54.60'N, 109°40.81'E, 745 m elev., 27 March 2021, Yao Shen leg., genitalia slide no. HSQ22162. ***Paratypes*.** China, **Hainan Prov.**, 3 ♂♂, other same data as holotype, paratype genitalia slide no. HSQ22164 ♂, paratype wing slide no. HSQ22004, HSQ22005; 1 ♀, Jianfengling Nature Reserve, 963 m elev., 24 June 2020, Ruo-Nan Xu & You Zeng leg.; 4 ♀♀, Qiongzhong Li and Miao Autonomous County, Shijie Reserve Station, 383 m elev., 26 March 2021, Yao Shen leg., paratype genitalia slide no. HSQ22168 ♀, HSQ22169 ♀; **Yunnan Prov.**, 1 ♂, Xishuangbanan Dai Autonomous Prefecture, 840 m elev., 23 May 2015, Man-Fei Tao leg., paratype genitalia slide no. HSQ22170.

#### Diagnosis.

This species is similar to *C.lubricosa* and *C.apicipicta* in appearance, but it can be differentiated by its darker body and wings, as well as the absence of the discoidal stigma on the hindwing, which is conspicuous in *C.lubricosa* and *C.apicipicta*. The wing venation of this species is somewhat different from that of *C.lubricosa*. The forewing of the latter has the R_S4_ slightly curved and close to R_S2+S3_ at the base, while the R_S4_ is straight and distant from R_S2+S3_ in this new species (Fig. 1A, B). Furthermore, the stalk length of M_1_ and Rs of the hindwing in this new species is longer than that of *C.lubricosa*. It also can be distinguished by the slender and extremely elongated phallus accompanied by an elongated, bent, needle-like cornutus; the elongated ductus bursae has a sclerotized longitudinal line approximately four-fifths of its length, and the corpus bursae is much shorter than the ductus bursae and has no signa. In *C.lubricosa* and *C.apicipicta*, the phallus is stout, and the former has a spicate cornutus and the latter has two fusiform cornuti; the ductus bursae has no longitudinal line, and the corpus bursae bears a pair of thin, band-like signa in these two species. The corpus bursae is much longer than the ductus bursae in *C.lubricosa*, and is almost as long as the ductus bursae in *C.apicipicta*.

#### Description.

***Adult*** (Fig. 3D). Body and wings dark brown, greyish. Forewing length 11.0–13.0 mm, wingspan 21.0–26.0 mm. Frons greyish brown, white laterally; vertex brown. Antenna brown, scape white ventrally. Labial palpi with first segment white, second and third segments dark brown. Maxillary palpi white, dark brown near apex. Patagium, tegula, and thorax dark brown. Fore and hind wings with terminal area pale grey. Forewing with orbicular and discoidal stigma black, sometimes indistinct; an elongated elliptical black patch at apex; a black line along terminal margin, discontinuous. Hindwing with a black line along terminal margin, discoidal stigma absent. Cilia brown, with a white basal line. Legs shiny white, epiphysis orange-yellow. Abdomen dark brown, pale grey ventrally.

***Male genitalia*** (Fig. 4E). Uncus long and thin, with the distal swollen and covered with minute setae, apex obtuse rounded and slightly concaved at middle. Valva oval, with sparse setae. Fibula thick, hooked apically. Saccus broad, strongly sclerotized. Phallus slender and extremely elongated, bent, with an elongated needle-like cornutus of nearly same length.

***Female genitalia*** (Fig. 4F). Apophyses anteriores ca 1.5 times as long as apophyses posteriores. Antrum weakly sclerotized. Ductus seminalis somewhat expanded near ductus bursae. Ductus bursae elongated, with a sclerotized longitudinal line approximately four-fifths of its length along one side. Corpus bursae oval, signa absent.

#### Etymology.

The specific name is derived from the Latin *aciculatus* for needle, in reference to the needle-like cornutus.

#### Distribution.

China (Hainan, Yunnan).

## ﻿Discussion

Kim et al. (2014) considered *Charitoprepeslubricosa* as a possible pest, but there has been no host reported for either of the two known species of the genus. The long, narrow, longitudinal signa are unusual in Spilomelinae. Besides *C.lubricosa* and *C.apicipicta*, the genus *Maruca* Walker, 1859 also have such signa, as well as a similar uncus. In *Agrioglypta* Meyrick, 1932, some species also have similar signa, but they are shorter and wider, and the other species have two rounded signa. Whether these special signa in *Charitoprepes* indicate a relationship to *Maruca* can only be made clear after a thorough phylogenetic study of the tribe Margaroniini, subfamily Spilomelinae.

## Supplementary Material

XML Treatment for Charitoprepes

XML Treatment for Charitoprepes lubricosa

XML Treatment for Charitoprepes apicipicta

XML Treatment for Charitoprepes aciculata
